# Correlation Study Between Abnormal Morphology of Meibomian Glands and Meibum in Patients With Dry Eye Disease Under *in vivo* Confocal Microscopy

**DOI:** 10.3389/fmed.2021.793338

**Published:** 2022-01-12

**Authors:** Qian Zheng, Yuanyuan Xue, Xiaowei Zhong, Guangwei Li, Weiyun Shi, Ting Wang

**Affiliations:** ^1^Department of Ophthalmology, Eye Hospital of Shandong First Medical University, Shandong First Medical University and Shandong Academy of Medical Sciences, Jinan, China; ^2^State Key Laboratory Cultivation Base, Shandong Provincial Key Laboratory of Ophthalmology, Shandong Eye Institute, Jinan, China; ^3^School of Ophthalmology, Shandong First Medical University, Jinan, China

**Keywords:** meibomian gland dysfunction, meibomian gland morphology, structure, function, abnormalities of meibum

## Abstract

**Objective:** To observe differences in meibomian gland morphology among patients with meibomian gland dysfunction (MGD) with liquid meibum, with solid meibum, and a non-MGD group by laser scanning *in vivo* confocal microscopy (IVCM), and then analyze the correlation between meibomian gland abnormalities and dry eye disease.

**Methods:** Twenty-one patients with MGD (42 eyes) with liquid meibum, 21 patients with MGD (38 eyes) with solid meibum, and 24 non-MGD patients (39 eyes) were enrolled in the study. IVCM examination and Schirmer II test were performed, and non-invasive tear-film breakup time (NIBUT) was measured.

**Results:** Data obtained from all the patients were normally distributed; therefore, one-way analysis of variance was performed. The meibomian gland opening diameter and gland opening area of the patients with MGD were greater than those of the non-MGD patients, and values of the liquid meibum group were greater than those of the solid meibum group (*F* = 17.96, *p* < 0.001; *F* = 8.84, *p* < 0.001, respectively). Due to changes in meibomian gland opening diameter and dilation of meibomian gland acini, the acinar longest diameter and unit area of the patients with MGD were also greater than those of the non-MGD patients, and the values of the solid meibum group were greater than those of the liquid meibum group (*F* = 36.52, *p* < 0.001; *F* = 27.81, *p* < 0.001, respectively). In the aspect of acinar shortest diameter, there was no difference among the three groups (*F* = 0.24, *P* > 0.05). Highest inflammatory cell density was observed in the solid meibum group, followed by the liquid meibum group, and the non-MGD group (*F* = 111.54, *p* < 0.001). Similarly, the results of the Schirmer II test and NIBUT showed that the condition of the patients with MGD in the solid meibum group was worst, followed by that of the liquid meibum group and the non-MGD group (*F* = 99.57, *p* < 0.001; *F* = 11.87, *p* < 0.001, respectively).

**Conclusions:** The different meibum in the patients with MGD is accompanied by different meibomian gland morphologies. Compared with the patients with liquid meibum, those with solid meibum have more obvious dilatation of meibomian glands under confocal microscopy and in these patients, the density of inflammatory cells among glands is higher, and the quality of tears is worse.

## Introduction

The prevalence of dry eyes is increasing day by day. In patients over age 40 years, the prevalence is as high as 75% (1), and the affected population is getting younger. Dry eye is a multifactorial disease of the ocular surface. It has been reported that the pathogenesis of dry eyes has a close relationship with meibomian gland dysfunction (MGD) (2, 3), which is the condition where the quantity and quality of lipid-rich secretion that is produced by meibomian glands (meibum) (4) are abnormal under influences of the eyes, whole body, medicine, external environment, and other factors (5). The meibum of patients with MGD may be clear and transparent liquid, cloudy liquid, cloudy and particulate fluid, or thick toothpaste-like solid (6). However, specific reasons for the different forms of meibum are still unclear. In this study, the patients with MGD are divided into two types: patients with liquid meibum and patients with solid meibum. In recent years, *in vivo* confocal microscopy (IVCM) has been performed to scan the structure of the ocular surface. The cell morphology of the cornea and meibomian gland can be determined. At cellular level, it has established a good connection between *in vitro* histology and *in vivo* ocular pathology (7, 8). In this study, IVCM was performed to detect the meibomian gland conditions of patients with different meibum qualities, and found that the gland morphology of patients with different meibum qualities was also different, which serves as an effective basis for in-depth research and treatment of MGD.

## Materials and Methods

### General Information

A total of 66 patients who were treated in Eye Hospital of Shandong First Medical University from June 2020 to August 2021 were involved. First of all, the patients received a slit-lamp examination for observation of meibomian gland orifice obstruction, followed by Schirmer II test and NIBUT for diagnosis of MGD. Then, IVCM examination was carried out. Finally, after meibomian gland massage, the patients with MGD whose meibum was clear or cloudy were divided into the liquid meibum group (21 cases, 42 eyes; seven males, 14 females), and the patients whose meibum was opaque and toothpaste-like were divided into the solid meibum group (21 cases, 38 eyes; 12 males and nine females). Those without dry eye symptoms, and with normal BUT and tear secretion were included in the non-MGD group (24 cases, 39 eyes; four males and 20 females). Massage was not performed on the non-MGD patients. The average age of the liquid meibum group was 58.8 ± 11.66 years, that of the solid group was 52.71 ± 18.5 years, and that of the normal subjects was 61.29 ± 14.87 years.

The inclusion criteria of the groups with MGD were as follows: (a) patient complained of dry eyes, foreign body sensation, burning sensation, eye fatigue, soreness, red jelly, and conscious blurred vision; (b) Schiermer II test < 10 mm, NIBUT < 10 s; (c) after meibomian gland massage, the meibum expressed is liquid or solid; and (d) participation in the research is voluntary.

Exclusion criteria were : patients (a) wearing contact lenses, or (b) having a history of eye trauma (acid-base burns), or (c) having had an eye surgery recently (9), or (d) with congenital abnormality or dysplasia of meibomian glands, or (e) with eye tattoos, (f) with immune system diseases such as Sjogren's syndrome (10), or (g) who have had long-term meibomian gland massage or fumigation, or (h) who have taken hormone drugs for a long time were excluded from the study.

The inclusion criteria for the non-MGD group were as follows: there is no eye dryness, foreign body sensation, and other discomforts in daily life; and at the same time, Schiermer II test >10 mm, and NIBUT >10 s.

This study was approved by the Ethics Committee of the Eye Hospital of Shandong First Medical University. All the procedures adhered to the tenets of the Declaration of Helsinki. Written informed consent was obtained from each patient. The trial was registered in the Chinese Clinical Trial Registry (registration number: ChicTR2100050900).

### Method

#### *In vivo* Confocal Microscopy (IVCM)

An Heidelberg hrt 3 (HRT 3) *in vivo* confocal microscope (Heidelberg Engineering, Heidelberg, Germany) was used to observe the morphological and structural characteristics of the meibomian glands at the center of the upper eyelid and eyelid margin after topical anesthesia (0.5% proparacaine hydrochloride) was instilled. The wavelength was 670 nm, field of view was 400 μm × 400 μm, resolution was 384 × 384 pixels, magnification was 800 times, and horizontal and vertical resolutions were both 1 μm. The center of the upper eyelid near the eyelid margin was scanned to observe meibomian gland opening, morphology of acini, and inflammatory cell infiltration.

#### Image Evaluation

In view of the fact that imaging of the meibomian gland acini and openings can be in different shapes when scanning planes are different, we selected 3–5 images with the best quality for analysis, including clear meibomian gland openings (at least 1 per image), acini (at least 5 per image), palpebral conjunctival cell structure, and inflammatory cells as many as possible. The diameter of all the meibomian gland openings, area of the meibomian gland openings, acinar longest diameter, acinar shortest diameter, and acinar unit area in the confocal microscopy images were measured with the Image J software. The fused acinar was calculated as one acinar and the incomplete acinar in the image was excluded. After the measurement, the authors calculated the area using the following formula: actual area = area × 400 × 400/(384 × 384) μm^2^. The number of inflammatory cells in the meibomian glands was measured using the Rostock Cornea Module. All the values were measured three times to get an average value.

#### Schirmer II Test

The lower eyelid of the patients was everted gently to instill topical anesthesia (0.5% proparacaine hydrochloride) and then, after a sterile filter paper strip was placed over the middle to lateral 1/3 of the lower eyelid, the patients were asked to close their eyes. The strip was removed after 5 min, and the length of tear wetting was measured.

#### Non-invasive Tear-Film Breakup Time (NIBUT)

Non-invasive tear-film breakup time (NIBUT) was evaluated with a Keratograph 5M ocular surface analyzer (10 times magnification). After two blinks, the patients were asked to focus on the target and keep the eyes open as long as possible to measure tear film breakup time.

#### Statistical Analysis

SPSS Version 23 was used for data analysis. The data obtained from patients in the three groups were normally distributed; therefore, one-way analysis of variance can be performed. *P* < 0.05 was regarded as statistically significant.

## Results

### General Results

The average age of the liquid meibum group, solid meibum group, and non-MGD group had no significant difference (58.15 ± 11.66 vs. 52.71 ± 18.5 vs. 61.29 ± 14.87) (*F* = 1.84, *P* > 0.05). Therefore, the influence of age difference on the results was avoided.

**Figure 1:**
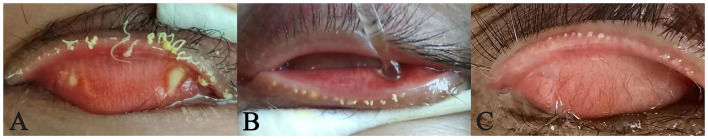
**(A)** Solid meibum and yellow oil embolism. **(B)** Solid meibum. **(C)** Oily liquid meibum.

According to the picture taken during meibomian glands massage, three of the 21 patients with solid meibum had light yellow oil embolism, which is the early state of a chalazion (Figure 1). The size of the toothpaste-like meibum in one patient was about 1 × 2 mm, and 0.5 × 1 mm in the other two patients.

### IVCM Results

#### IVCM Meibomian Gland Opening Examination

**Figure 2:**
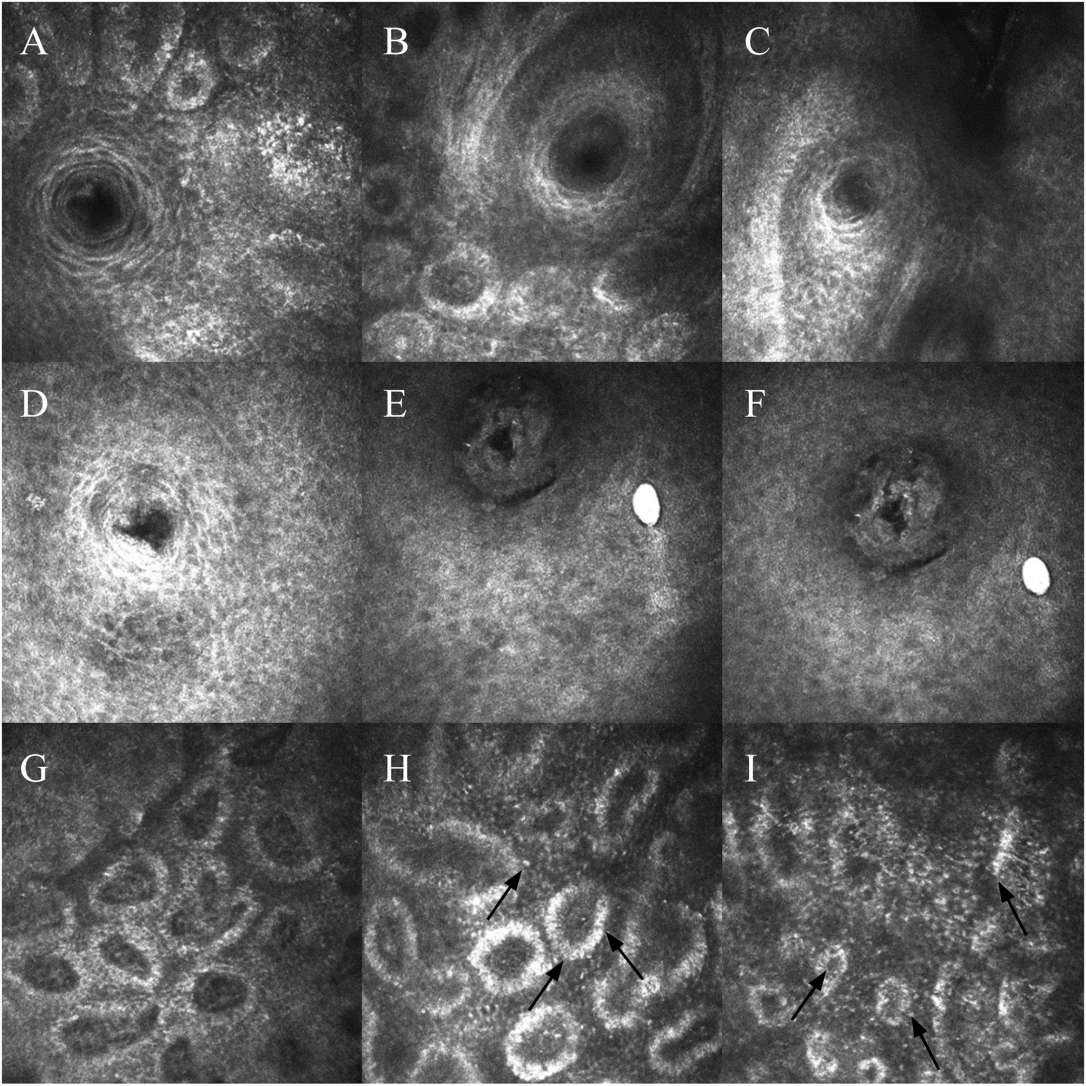
**(A–C)** Meibomian gland openings of patients with liquid meibum. Cells surrounding the opening are arranged neatly, with regular borders. **(D–F)** Meibomian gland openings of patients with solid meibum. Morphology has changed, and the glands become blocked. The morphology of cells surrounding the opening has changed, and the arrangement is looser. **(G)** Image of a patient with liquid meibum. The glands have a certain degree of dilatation. **(H,I)** Images of patients with solid meibum. There is more dilatation and fusion between the acini. Black arrows indicate inflammatory cells.

In the patients with liquid meibum, the meibomian gland openings were round or oval, or nearly circular, surrounded by round and oval flat cells, and loosely arranged with irregular borders, similar to the surrounding conjunctival epithelial cells. The morphology of meibomian gland opening cells in the patients with solid meibum was different. The cells were irregularly long or diamond-shaped and more loosely arranged, and the number of goblet cells was increased. There was a small amount of scattered dendritic cells around, and more fibrotic tissues existed. The surrounding conjunctival epithelial cells had increased size and were arranged more irregularly, accumulating around the meibomian gland acini (Figure 2). A certain degree of blockage existed in the meibomian gland openings of patients with solid meibum. Compared with the two groups mentioned above, the meibomian gland of the non-MGD patients was round in shape, and had regular boundary and orderly cell arrangement. According to the results calculated with SPSS Version 23, the meibomian gland opening diameter and gland opening area of the patients with MGD were greater than those of the non-MGD patients, and the values of the liquid meibum group were greater than those of the solid meibum group. Statistical analysis of the opening diameter and opening area of the meibomian gland openings showed that there were statistically significant differences among the three groups. There were also statistical differences between any two groups (opening diameter: 39.47 ± 9.57 vs. 34.8 ± 9.98 vs. 26.21 ± 10.6; opening area: 1,861.47 ± 910.4 vs. 1,517.23 ± 713.12 vs. 1,188.96 ± 437.13) (*F* = 17.96, *p* < 0.001; *F* = 8.84, *p* < 0.001, respectively).

#### Meibomian Gland Acini Examination Under IVCM

The acini of the meibomian gland were mostly round, oval, or irregular because of fusion, and each acinar unit was surrounded by cubic acinar cells. There was a connective tissue among the acini. Compared with the acini of patients with liquid meibum, the acini of patients with solid meibum had more obvious acinar dilatation and more fusion phenomena. Gland acinar shortest diameter in patients with liquid meibum, in patients with solid, and in the non-MGD group had no statistically significant difference (29.26 ± 8.23 vs. 28.04 ± 10.28 vs. 29.49 ± 10.6) (*F* = 0.24, *p* > 0.05). The acinar longest diameter and unit area of the patients with MGD were also greater than those of the non-MGD patients, and the values of the solid meibum group were greater than those of the liquid meibum group, with statistically significant differences. There were also statistical differences between any two groups (132.86 ±3 7.69 vs. 100.65 ± 25.54 vs. 77.07 ± 20.81; 4,804.49 ± 1,711.25 vs. 3,229.97 ± 1,108.35 vs. 2,639.89 ± 1,055.02) (*F* = 36.52, *p* < 0.001; *F* = 27.81, *p* < 0.001) (Table 1).

**Table 1:** Parameters of the meibomian glands under a confocal microscope.

**Group**	**Meibomian gland opening diameter /μm**	**Meibomian gland opening area /μm^**2**^**	**Acinar longest diameter /μm**	**Acinar shortest diameter /μm**	**Acinar unit area/μm^**2**^**	**Density of inflammatory cells/mm^**2**^**
Liquid meibum group	39.47 ± 9.57	1,861.47 ± 910.40	100.65 ± 25.54	29.26 ± 8.23	3,229.97 ± 1,108.35	395.11 ± 60.09
Solid meibum group	34.80 ± 9.98	1,517.23 ± 713.12	132.86 ± 37.69	28.04 ± 10.28	4,804.49 ± 1,711.25	583.50 ± 103.42
Non-MGD group	26.21 ± 10.60	1,188.96 ± 437.13	77.07 ± 20.81	29.49 ± 10.6	2,639.89 ± 1,055.02	308.30 ± 80.78
F	17.96	8.84	36.52	0.24	27.81	111.54
P	<0.001	<0.001	<0.001	0.78	<0.001	<0.001

#### Inflammatory Cells Around the Meibomian Glands Under IVCM

The missing part of meibomian glands in the infrared imaging was manifested as atrophy and loss of meibomian acini under IVCM, or the acinar structure was replaced by a large number of cord- and grid-like structures and inflammatory cells. In the patients with MGD, there was often inflammatory cell infiltration among the acini. Highest inflammatory cell density was observed in the solid meibum group, followed by the liquid meibum group and the non-MGD group, with statistically significant differences. There were also statistical differences between any two groups (583.5 ± 103.42 vs. 395.11 ± 60.09 vs. 308.3 ± 80.78) (*F* = 111.54, *p* < 0.001) (Table 1 and Figure 1).

### Schirmer II Test

SPSS Version 23 was used to analyze the data obtained from the patients with solid meibum (21 cases, 42 eyes), patients with liquid meibum (21 cases, 38 eyes), and the non-MGD group (24 cases, 39 eyes). The parameter of the non-MGD group was the longest. The Schirmer II test results of the patients with liquid meibum were greater than those of the patients with solid meibum. The differences among the three groups were statistically significant. There were also statistical differences between any two groups (12.43 ± 2.76 vs. 6.6 ± 3.49 vs. 3.76 ± 1.56) (*F* = 99.57, *p* < 0.001) (Table 2).

**Table 2:** Non-invasive tear breakup time and Schirmer II test results.

**Group**	**Non-invasive tear break-up time (s)**	**Schirmer II test**
Liquid meibum group	9.30 ± 4.83	6.60 ± 3.49
Solid meibum group	6.73 ± 3.69	3.76 ± 1.56
Non-MGD group	11.52 ± 4.27	12.43 ± 2.76
F	11.87	99.57
P	<0.001	<0.001

### Non-invasive Tear-Film Breakup Time (NIBUT)

The NIBUT in each group obtained with the Keratograph 5M was analyzed with SPSS Version 23. The NIBUT of the patients with liquid meibum was longer than that of the patients with solid meibum, and the time of the non-MGD group was longest, with statistically significant differences. There were also statistical differences between any two groups (9.3 ± 4.83 vs. 6.73 ± 3.69 vs. 12.43 ± 2.76) (*F* = 11.87, *p* < 0.001) (Table 2).

## Discussion

Meibomian glands, first described by Heinrich Meibom, a German anatomist, are the tarsal glands of the eyelids located between the massive connective tissue and elastic fibers (11), producing meibum to lubricate the surface of the lids. Meibum is the primary component of the surface lipid layer of the tear film, which lowers tear film surface tension to increase stability (12). MGD caused by abnormal secretion of meibum is the main cause of hyperevaporative dry eyes (13). Previous clinical studies have reported that there is a certain correlation between decline of meibomian gland function and morphological changes, but that exceptions also exist (14, 15). When several glands become blocked in an early stage, although the morphology and structure of the meibomian glands are normal, the meibum that is delivered to the ocular surface is reduced or completely inhibited. Under this circumstance, the function of the meibomian glands is weak. When meibomian gland atrophy appears, the morphology changes, and function decreases. At present, there are only few studies on the relationship between meibum status and morphology of the meibomian glands in patients with MGD. In this study, the relationship between meibum status and abnormal morphology of meibomian glands was analyzed by confocal microscopy for the first time.

The status of lipid secretion of the meibomian glands can roughly reflect the degree of meibomian gland dysfunction. According to the study conducted by Eom et al., the meibum expression was evaluated as follows: grade 1: cloudy meibum expressed with mild pressure; grade 2: cloudy meibum expressed with more than moderate pressure; and grade 3: toothpaste-like meibum (16). The higher the score, the more severe the dysfunction. That is, when the meibum score reaches grade 3, the meibomian gland acini have been extremely dilated, which is manifested by obvious increase in longest diameter and acinar area. Excessive dilation of ducts will reverse the pressure to the acini, which will result in acinar damage, meibomian gland atrophy, and destruction of acinar structures.

Conjunctival epithelial cells and subepithelial connective tissue layers can be observed by IVCM, among which are openings of the meibomian glands. In this study, meibomian gland opening diameter and area were studied in detail for the first time. Compared with the non-MGD group, the morphology of the meibomian glands in the MGD groups was changed. Consistent with the research findings by Yang et al., the results of this study showed that the meibomian gland openings in patients with liquid meibum were round or oval and surrounded by loosely-arranged flat cells (17), and high-reflective oil can occasionally be seen. While in the patients with solid meibum, the meibomian gland openings had obvious keratinization, damage, deformation, and unevenness, and were surrounded by increased inflammatory cells. The authors explored the reasons: heavily influenced by risk factors such as blockage, edema, inflammation, environmental stress, and stem cell aging (18), hyperkeratosis of the epithelium, and stagnant secretion of the meibum happened. The inability of the meibum to secrete normally promotes growth of bacteria, leading to the release of bacterial lipase secretion, which can break down the meibum. The increase of free fatty acids can change the viscosity of the meibum, which is originally clear liquid or oily, to granular or toothpaste-like solid secretions further, and consequently increases the difficulty of releasing meibum (19). In addition, after measuring the parameters of the patients included, the authors found that when suffering from MGD, the opening of the meibomian gland was dilated, but that the dilation in the patients with solid meibum was not as obvious as that in patients in the liquid meibum group, which was also unfavorable for releasing of solid meibum. The gradually increased pressure inside the blocked ducts led to the compensatory dilation of the acini and more fusions. The acinar longest diameter increased significantly, and so did the unit area (2). In turn, these processes promoted the inflammation and hyperkeratosis of the meibomian glands (20), which intensified the deformation and atrophy of the meibomian gland openings in patients with solid meibum.

In healthy people, there is occasional infiltration of inflammatory cells in the acini and interstitium, about 300 cells/mm^2^. In this study, the density of inflammatory cells in the patients with MGD was greater than that of the non-MGD patients, and the value of the solid meibum group was greater than that of the liquid meibum group (583.5 ± 103.42) cells/mm^2^ vs. (395.11 ± 60.09) cells/mm^2^, suggesting that when the condition got worse, the inflammation got more severe. In addition to the mentioned interactions among the abnormal meibum, etiology, and morphology of the meibomian glands, the density of inflammatory cells indicates the status of the meibum and development stage of the disease (21). Yang et al. established an animal model of MGD in mice and found that after Freund's adjuvant injection, ductal epithelial thickening, acinar cell atrophy, and increased inflammatory cell infiltration were observed. In the solid meibum group, although the meibomian acini had not fully developed to an atrophic state, there was a large number of scattered inflammatory cells among the acini and interstitium. The number of inflammatory cells in the patients with liquid meibum was less than that of the patients with solid meibum, but there was an increase in the number in the patients from both groups, which further proved that inflammation is a typical symptom of MGD. As the meibomian glands were continually in a microenvironment with inflammatory cell infiltration, stem cells that can undergo meiosis were senescent, and the morphology of the glands was also destroyed. In patients with severe symptoms, meibomian gland openings were more uneven, and cell arrangement was looser, with more fibrotic tissues. When meibomian gland dysfunction develops to a certain stage, inflammatory factors can cause corneal nerve damage and symptoms such as burning and tingling will become more obvious (22).

The results of the Schirmer II test showed the tear secretion condition. The amount of tear secretion in the solid meibum group, which was 3.76 ± 1.56 mm, was significantly lower than the 6.6 ± 3.49 mm in the liquid meibum group. Previous studies have confirmed that 10% of tears are discharged through evaporation, and that 90% are discharged through tear ducts (23). When the function of the meibomian glands is impaired, the tear film will also be affected, and the discharge of tears will mainly rely on evaporation. When lipid secretion is insufficient, the meibum cannot be distributed evenly on the surface of the tear film by blinking; therefore, the tear film is unstable, and tear evaporation is strong, leading to decrease in tear secretion. The tear secretion of patients with liquid meibum is close to that of healthy people, but the tear secretion of patients with solid meibum is obviously insufficient, which is also reflected in the following NIBUT research.

Non-invasive tear-film breakup time (NIBUT) is an objective test to evaluate the stability of the tear film. NIBUT > 10 s is generally considered as normal, while < 10 s is unstable. The physiological tear film is divided into an amucoaqueous-gel layer and a lipid layer, and the lipid layer is divided into polar and non-polar sublayers. The stability of the tear film depends on the function of mucoaqueous gel and lipid layers in the tear film. Meibum can reduce the surface tension of tears to facilitate the absorption of water into the tear film. As a result, the aqueous phase is thickened, and the tear film is more stable (24, 25). Lack of mucin in tear fluid indicates severe damage or loss of goblet cells. Changes in the lipid composition of the tear film can also contribute to increased instability of tears. An ocular surface analyzer can accurately record the specific location and time of tear film rupture in the form of corneal topography (26). First tear film rupture time is usually used as an indicator to evaluate the condition of MGD. In this study, the first tear film rupture time in the solid meibum group was shorter than that in the liquid meibum group (*F* = 11.87, *p* < 0.001), indicating that the tear film of the patients with solid meibum was more unstable. Therefore, the eyes were more likely to develop into dry eyes with excessive evaporation. When tear osmotic pressure continues to increase, it is more likely to result in ocular surface epithelial damage and lead to activation of inflammatory cells to release inflammatory factors (27), which, at the same time, indirectly explains why the patients with solid meibum have more inflammatory cells than the patients with liquid meibum.

From the perspective of the composition of meibum, lipid composition changes increase order degree and melting point. In patients with MGD, the viscosity of lipids increased because of composition changes in the meibomian glands. Meibum is divided into polar and -polar lipids, and polar lipids play an extremely important role in tear film stability (28). Sphingolipids, which are membrane lipids that exist in almost all cell types (29), account for 30% of polar lipids in meibomian glands and play a dominant role in cell signal transduction, inflammation, and apoptosis (30). Studies have shown that the clinical symptoms of patients with MGD are related to changes in the composition of sphingolipids, and that pro-apoptotic substances increase after the composition changes. We can boldly make a guess that under stimulation of pro-apoptotic substances, meibomian gland basal cells and the ductal epithelium of patients with solid meibum suffer from hyperkeratosis and apoptosis, which makes it difficult for the meibum to enter the central duct and accelerates changes in acinar morphology in patients with MGD. Therefore, with IVCM, we observed changes in the morphology of meibomian gland opening cells and increase in inflammatory cells. Different meibum status indicates different meibomian gland morphology, and different stages of inflammation, which are ultimately associated with clinical symptoms.

While conducting the study, it was expected that the number of patients with solid meibum would be higher. However, with the improvement of living standards in modern society, most patients seek medical attention in the early stage of MGD; therefore, fewer patients reach the clinical stage with solid meibum, so the solid meibum samples included in the study were limited. We will include more patients for further in-depth research in the future. In addition, most of the included patients were middle-aged and elderly people, especially in the non-MGD group, which may have an impact on the overall data (31).

With confocal microscopy, this study observed the meibomian gland morphology of patients with different meibum at the microscopic cellular level and gained a clearer understanding of the specific morphology and inflammation in patients with different meibum. The objective parameters obtained by observing different meibum status by confocal microscopy will help to divide the different stages of MGD in the future and guide the diagnosis and treatment of patients with MGD.

## Data Availability Statement

The original contributions presented in the study are included in the article/supplementary material, further inquiries can be directed to the corresponding author/s.

## Ethics Statement

The studies involving human participants were reviewed and approved by the Ethics Committee of the Eye Hospital of Shandong First Medical University. The patients/participants provided their written informed consent to participate in this study.

## Author Contributions

TW, WS, and QZ contributed to the conception and design of the study and performed the statistical analysis. TW, QZ, YX, XZ, and GL organized the database. TW and QZ wrote the first draft of the manuscript. TW and WS reviewed and revised the manuscript. All the authors contributed to manuscript revision and read and approved the submitted version.

## Funding

This study was supported by the National Natural Science Foundation Regional Innovation and Development Joint Fund [U20A20386], Natural Science Foundation of Shandong Province [ZR2019MH135 and ZR2019ZD37], Young Taishan Scholars [tsqn201909188], and Academic Promotion Programme of Shandong First Medical University [2020RC004].

## Conflict of Interest

The authors declare that the research was conducted in the absence of any commercial or financial relationships that could be construed as a potential conflict of interest.

## Publisher's Note

All claims expressed in this article are solely those of the authors and do not necessarily represent those of their affiliated organizations, or those of the publisher, the editors and the reviewers. Any product that may be evaluated in this article, or claim that may be made by its manufacturer, is not guaranteed or endorsed by the publisher.
